# Functional morphology of antennae and sensilla of *Hippodamia variegata* (Coleoptera: Coccinellidae)

**DOI:** 10.1371/journal.pone.0237452

**Published:** 2020-08-07

**Authors:** Ya-Nan Hao, Yuan-Xing Sun, Chang-Zhong Liu

**Affiliations:** Biocontrol Engineering Laboratory of Crop Diseases and Pests of Gansu Province, College of Plant Protection, Gansu Agricultural University, Lanzhou, China; University of São Paulo, BRAZIL

## Abstract

The lady beetle *Hippodamia variegata* is an important biocontrol agent of many aphids. In this study, the fine morphology of antennae as well as the typology, morphology and distribution of antennal sensilla were comprehensively examined by scanning electron microscopy. The antennal morphology of female and male are similar and consist of the scape, pedicel, and nine flagellomeres. No significant difference was detected in the length of each segment between two sexes, while the male antennae are much stronger than females. In total, six types of sensilla can be defined on antenna, including Böhm bristle, sensilla chaetica (with three subtypes), sensilla basiconica (with three subtypes), sensilla trichodea, sensilla placodea and sensilla coeloconica. It is worth noting that sensilla chaetica III distributed only on the fixed position of male antennae. In addition, the functional morphology of antennae of *H*. *variegata* were compared with other lady beetles from multiple perspectives. Specially, the function of sensilla were also discussed according to their morphology, location and information from previous studies.

## Introduction

Insect antennae are segmented appendages that are well-equipped with a wide variety of sensilla undertaking olfactory, tactile or gustatory function [1]. Although sensilla are distributed all over the insect body [2,3], those located on antenna play the most important roles and thus make antenna the primary peripheral olfactory system for most insects [4–7]. Depending on these sensilla, antennae play critical roles in host recognition and location, mating and other behaviors in their entire lifespan [5,8–11]. Antennae may vary considerably in length, morphology, number of segmentation and the size of individual segment in different insects or different sexes of the same species. Moreover, the incidence, density, types and distribution of sensilla and other aspects that closely related to their function may also greatly differ among different species [12–16]. These remarkable differences of sensory equipment in antennae have many potential values for taxonomic and ecological research and in behavior analyses [17–19].

The lady beetle *Hippodamia variegata* (Goeze, 1777) (Coleoptera: Coccinellidae: Coccinellinae) is distributed worldwide [20–26], and has been proved to be a useful generalist predator of many aphids [25,27], noctuid larvae [28], leafhoppers [22] and psyllids [20]. Many previous studies have been conducted on the biological and ecological characteristics of *H*. *variegata* (e.g. life table parameters, phenological characteristics and functional response to different preys) to illustrate its biocontrol potential [26,29–33]. However, limited data has been published to concentrate on their morphological features, especially antennae, one of the prime sensory organs. The morphological studies may reveal their unique functions through the micro-observation of the antennal structure as well as the types and number of sensilla. Former studies have revealed that the antennae of ladybeetles are clavated [34] and consist of scape, pedicel and several flagellomeres, while the number of flagellomeres and types of sensilla greatly varied among different species [34–37]. The primary aim of this study was to investigate the fine morphology of the antenna and, more importantly, the types and distribution of antennal sensilla of *H*. *variegata*. Besides, the putative function of these sensilla were discussed. The data from this study could provide vital cues for further understanding the mechanisms of prey-foraging of this lady beetle, and thus contributed to the more effective use of them in pest management.

## Materials and methods

### Insect collection

*Hippodamia variegata* adults were collected on the campus of Gansu Agricultural University in Lanzhou, Gansu Province, China (36° 03′ N, 103° 40′ E) in June 2019, and then reared in a bioclimatic chamber (25°C, 70% RH, 16:8 L:D) with pea aphids. The female and male adults were respectively preserved in 70% ethanol, and stored at 4°C for observation with scanning electron microscopy.

### Scanning electron microscopy

The specimens were rinsed twice with 70% ethanol using an ultrasonic cleaner (KQ118, Kunshan, China), twenty seconds for each time. And then, samples were dehydrated in agraded series of 80%, 90% ethanol for 20 min each and 99.9% ethanol for 30 min twice before being transferred to a mixed solution of ethanol and tertbutanol (3:1, 1:1, and 1:3, by volume) for 15 min each, and finally dipped into 100% tert-butanol for 30 min. After that, the samples were dried with a freeze-drier (VFD-21S, SHINKKUVD, Japan) for 3 h. The dried antennae were then separated from bodies and mounted on the aluminum stubs under a stereomicroscope with double-sided copper sticky tape and coated with gold/palladium (40/60) in a high-resolution sputter coater (MSP-1S, Hitachi, Tokyo, Japan). Fine morphology of four female and four male antennae were examined with a Nova Nano 450 SEM (FEI, America) operated at 15 kV.

### Image processing and morphometric measurement

Photographs of SEMs were processed and measured after being imported into Adobe Photoshop CS6 (Adobe Systems, San Jose, CA, USA). Statistical analyses were executed using SPSS 19.0 (SPSS, Chicago, IL, USA). The differences of lengths and widths of each antennal segment between female and male were compared with a paired two-tailed, Student *t*-test (*p* < 0.05). The sensilla were classified according to their external morphology, length, and distribution. To characterize the sensilla, we used the nomenclature proposed by Altner and Prillinger [38] with more specialized nomenclature from Chi [35].

## Results

### Gross morphology of antennae

**Fig 1:**
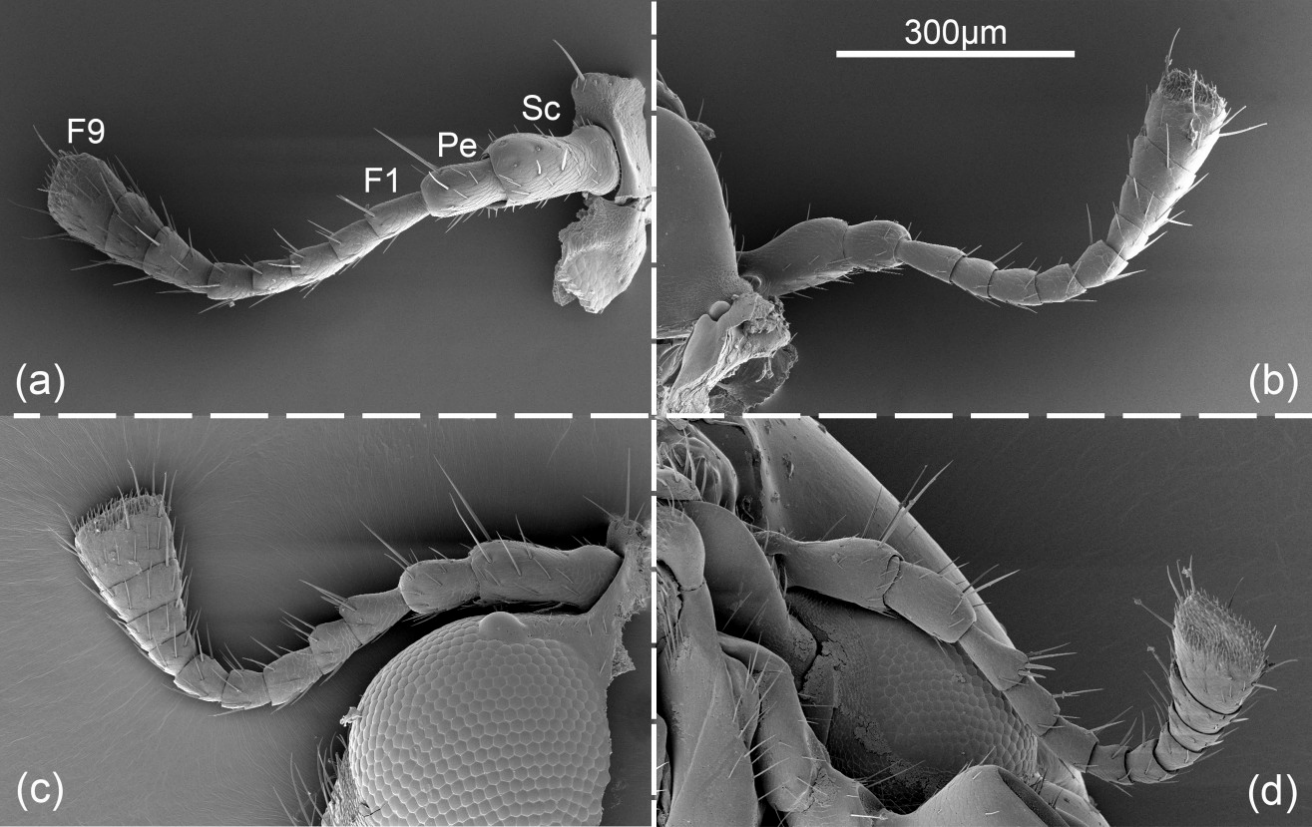
*Hippodamia variegata*; Scanning electron micrographs of the antennae. (a) Dorsal surface of female antenna. (b) Ventral surface of female antenna. (c) Dorsal surface of male antenna. (d) Ventral surface of male antenna. Sc, scape; Pe, pedicel; F1-F9, the first to ninth flagellomere.

**Fig 2:**
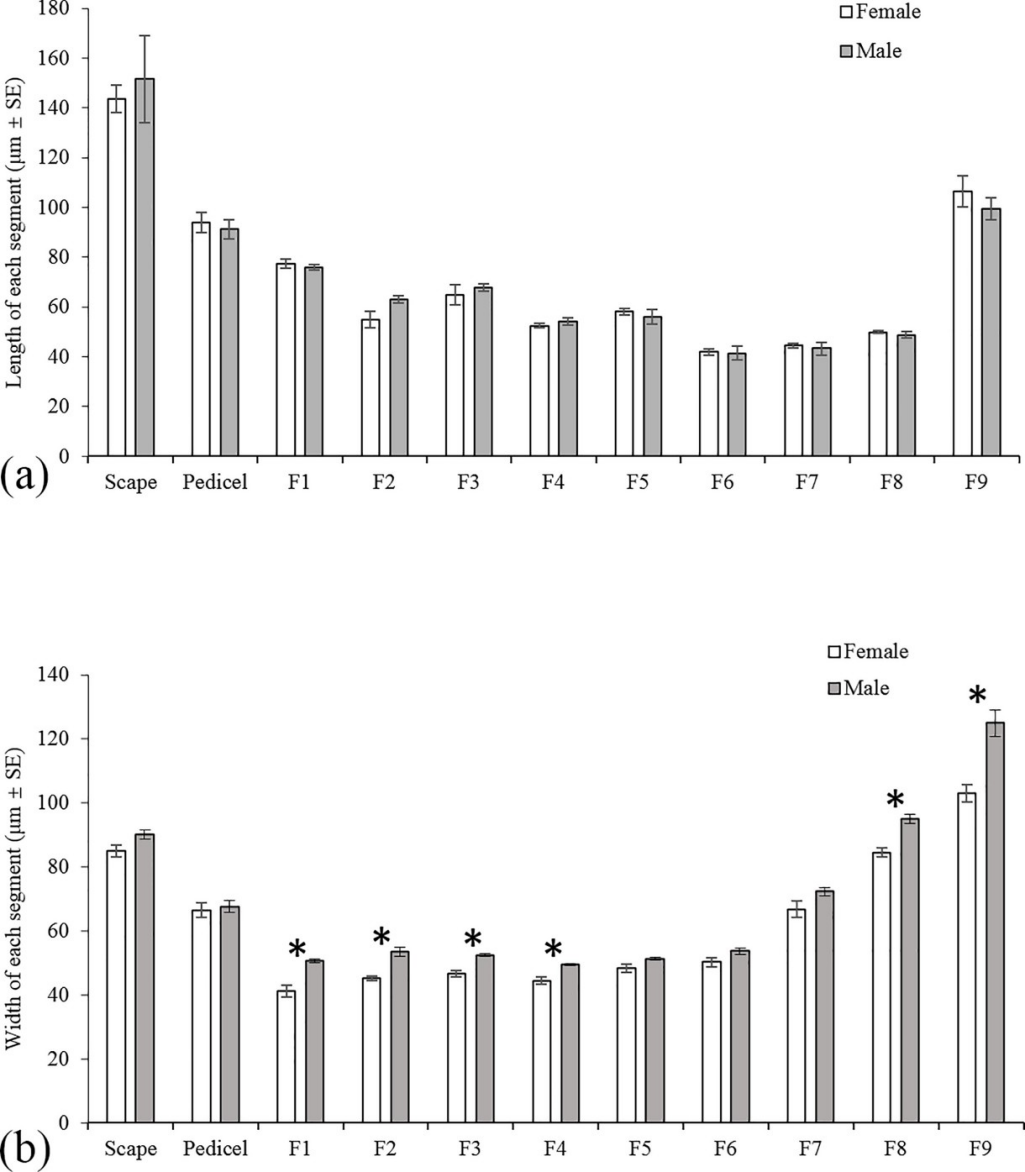
The length and width of each antennal segment in both sexes of *Hippodamia variegata*. (a) The length of each antennal segment. (b) Width of each antennal segment. Asterisk indicates significant difference in 0.05 level, values shown are the mean ± standard error.

The antennae of *H*. *variegata* are hammer-like and located in front of compound eyes at the dorsolateral corners of the frons. The antennal morphology of female and male are similar and consist of the scape, pedicel, and nine flagellomeres (F1-F9) (Fig 1). All segments are cylindrical, and each flagellomere is gradually thicker from base to tip (Fig 1). The total length of female antennae is slightly less than male (Female: 788.26 ± 19.15 μm, Male: 793.53 ± 26.04 μm). For both sexes, scape is the longest segment in antennae (Female: 143.47 μm, Male: 151.59 μm), which is followed by F9 (Female: 106.53 μm, Male: 99.57 μm). There is no significant difference between females and males on the length of each segment (*t* = -2.439–0.960, *p* = 0.059–0.886) (Fig 2A). However, all segments of male antennae are relatively wider than those of female (Fig 2B), and the significant levels reached in F1, F2, F3, F4, F8 and F9 (F1: *t* = -5.118, *p* = 0.002; F2: *t* = -5.158, *p* = 0.002; F3: *t* = -5.217, *p* = 0.002; F4: *t* = -3.934, *p* = 0.008; F8: *t* = -5.239, *p* = 0.002; F9: *t* = -4.388, *p* = 0.005).

The surface of antennae is scaly with various kinds of sensilla distributed on it. In total, six types of sensilla can be defined, including Böhm bristle (Bb), sensilla chaetica (Sch), sensilla basiconica (Sb), sensilla trichodea (Str), sensilla placodea (Sp) and sensilla coeloconica (Sco), among which Sch and Sb can be further divided into three subtypes, respectively (Table 1).

**Table 1:** Morphological characteristics and distribution of the antennal sensilla of *Hippodamia variegata*.

	Shape	Socket	Surface	Length (μm)	Basal diameter (μm)	Distribution
Bb	Peg	Concave	Smooth	4.15–15.97	1.69±0.13	Scape, pedicel
Sch1	peg	Concave	Grooved	9.98–39.85	2.29±0.13	All segments
Sch2	Hair, peg	Concave	Grooved	52.97–103.6	4.19±0.36	All segments
Sch3	Peg	Concave	Grooved	31.58–45.05	5.79±0.20	F1 of male
Sb1	Peg	Convex	Smooth	4.38–7.53	1.96±0.06	F8, F9
Sb2	Peg	Convex	Smooth	7.55–8.35	2.18±0.11	F9
Sb3	Peg	Convex	Smooth	11.32–15.31	2.11±0.04	F8, F9
Str	Hair	Convex	Smooth	8.23–11.39	1.31±0.07	F9
Sp	Round	Convex	Smooth		1.20±0.01	Scape of female
Sco	Round	Concave			0.81±0.06	All segments

Bb = Böhm bristle; Sch1-3 = sensilla chaetica I-III; Sb1-3 = sensilla basiconica I-III; Str = sensilla trichodea; Sp = sensilla placodea; Sco = sensilla coeloconica. F1-9 = the first to ninth flagellomere. Date of basal diameter are Mean ± SE.

**Fig 3:**
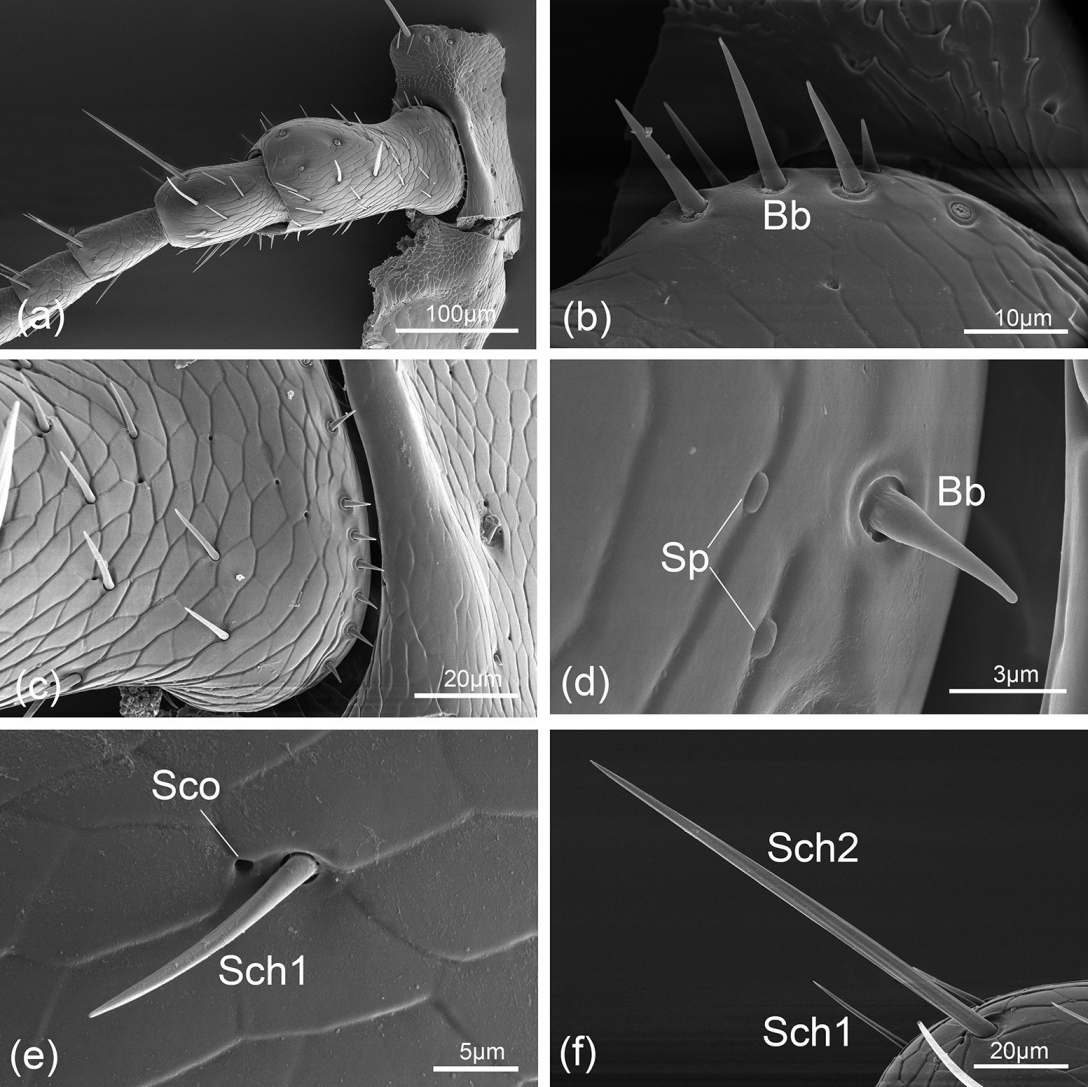
*Hippodamia variegata*; scanning electron micrographs of the dorsal surface of female antennae and the sensilla. (a) Morphology of scape, pedicel and the first two flagellomeres. (b) Böhm bristle (Bb). (c) Enlarged views of the basal part of scape showing the scaly surface and different types of sensilla. (d) Böhm bristle (Bb) and sensilla placodea (Sp). (e) Sensilla chaetica I (Sch1) and sensilla coeloconica (Sco). (f) Sensilla chaetica I (Sch1) and sensilla chaetica II (Sch2).

**Fig 4:**
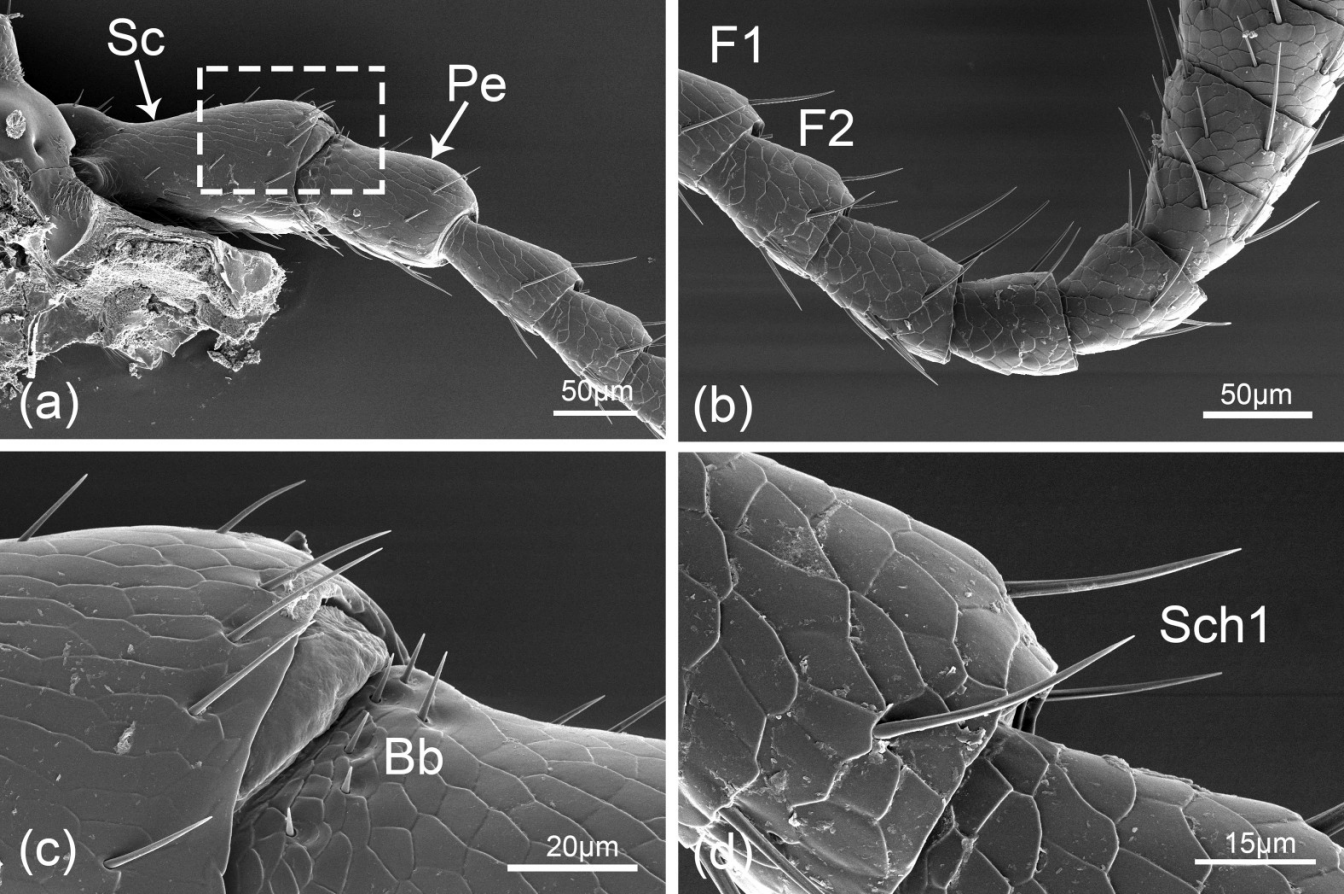
*Hippodamia variegata*; scanning electron micrographs of the ventral surface of female antennae and the sensilla. (a) Morphology of scape (Sc), pedicel (Pe) and the first two flagellomeres. (b) Ventral surface of flagellum. F1, the first flagellomere. (c) The intersegmental area between scape and pedicel showing Böhm bristle (Bb). (d) Sensilla chaetica I (Sch1) on flagellum.

The dumbbell-like scape is the longest segment in antennae, which narrows in the middle part (Fig 3A). This segment is relatively longer and wider in male than in female, but not differed significantly (Fig 2A and 2B). Several types sensilla distributed on this segment, including Bb (Fig 3B), Sch1 (Fig 3E), Sch2 (Fig 3F), Sp (Fig 3D) and Sco (Fig 3E). The sensilla on dorsal surface of scape are more abundant than those on ventral surface both in male and female (Figs 3A and 4A).

**Fig 5:**
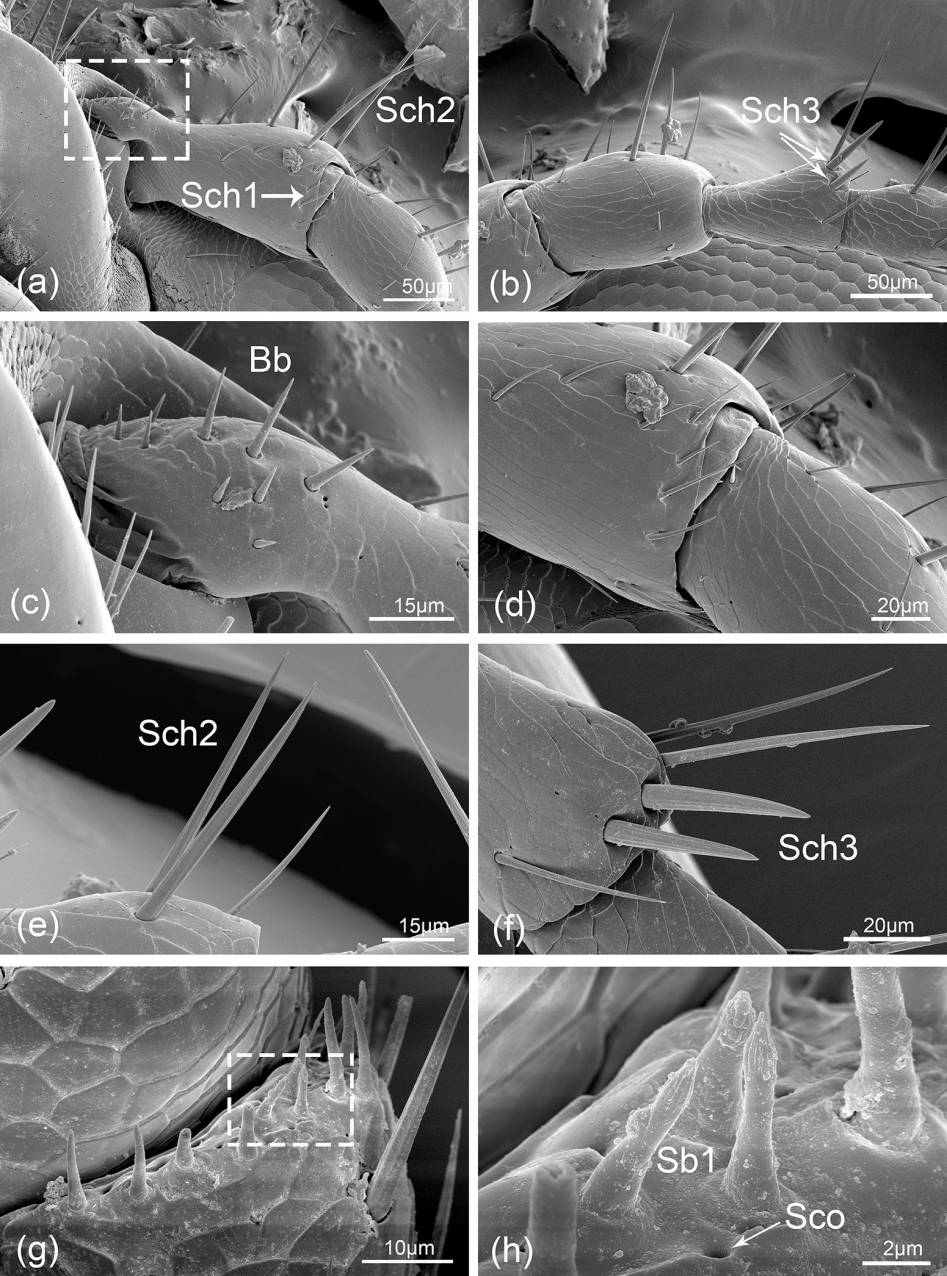
*Hippodamia variegata*; scanning electron micrographs of the ventral surface of male antennae and the sensilla. (a) Enlarged view of scape showing sensilla chaetica I (Sch1) and sensilla chaetica II (Sch2). (b) Enlarged view of pedicel and the first flagellomere showing sensilla chaetica III (Sch3). (c) Enlarged view of the white dashed box in (a) showing Böhm bristle (Bb). (d) The intersegmental area between scape and pedicel. (e) Sensilla chaetica II (Sch2). (f) Sensilla chaetica III (Sch3). (g) The terminal part of the eighth flagellomere showing various types of sensilla. (h) Enlarged view of the white dashed box in (g) showing sensilla basiconica I (Sb1) and sensilla coeloconica (Sco).

Similar to scape, more sensilla were found on dorsal surface than on ventral surface, which are consisted of Sch1 (Fig 3E), Sch2 (Fig 3F), Sco (Fig 3E) and Bb (Figs 4C and 5C). There is also no difference between female and male in sensilla types and quantity (Figs 3A and 4A).

**Fig 6:**
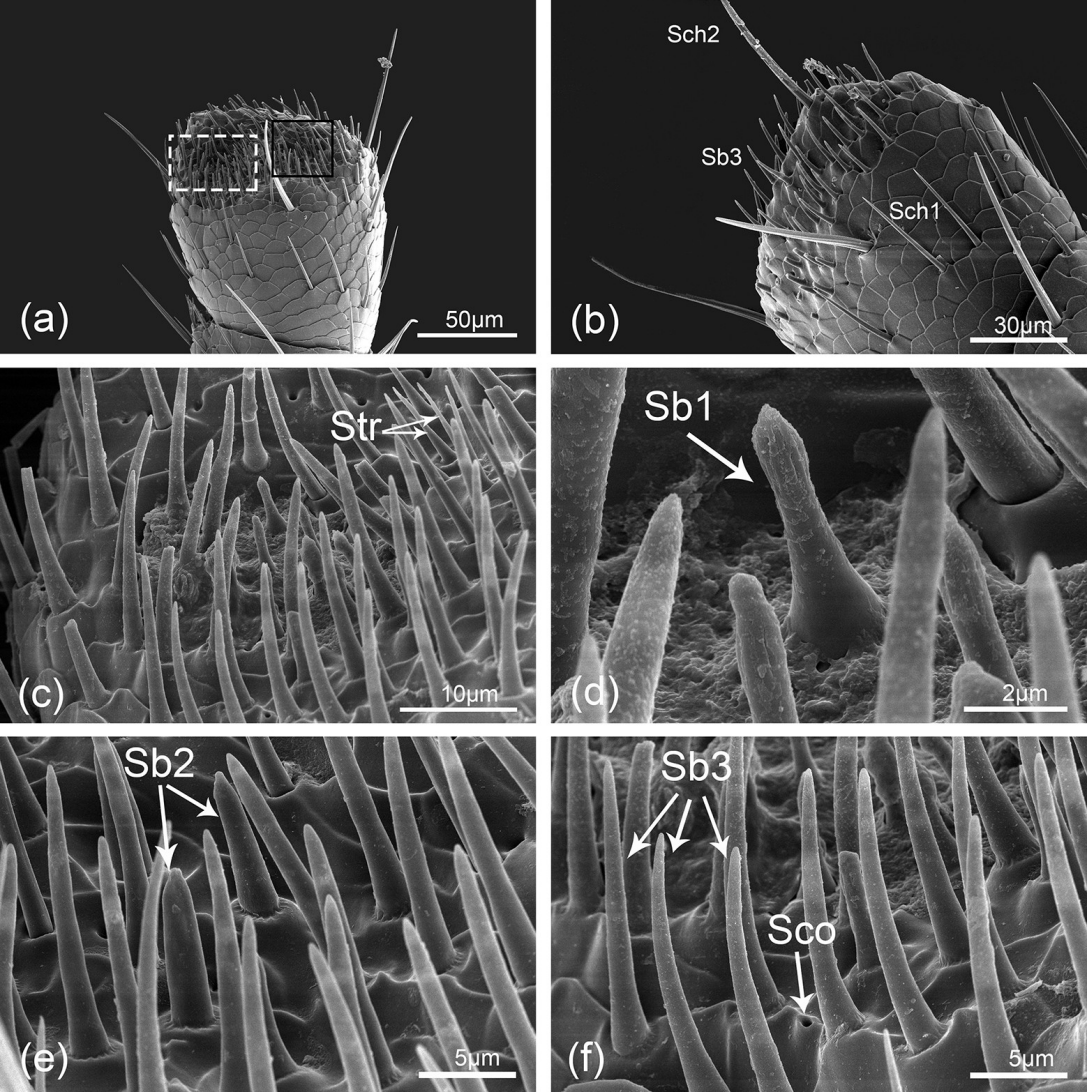
*Hippodamia variegata*; Scanning electron micrographs of the sensilla on the last flagellomere of female. (a) Ventral surface of the last flagellomere. (b) Dorsal surface of the last flagellomere showing sensilla chaetica I (Sch1), sensilla chaetica II (Sch2) and sensilla basiconica III (Sb3). (c) Enlarged view of the white dashed box in (a) showing sensilla trichodea (Str) and other sensilla. (d) Sensilla basiconica I (Sb1). (e) Sensilla basiconica II (Sb2). (f) Sensilla basiconica III (Sb3) and sensilla coeloconica (Sco).

Among the nine flagellomeres, the last flagellomere (F9) is the longest and widest one in both sexes. The length of each flagellomeres is similar in the two sexes (Fig 2A), but the width of F1, F2, F3, F4, F8 and F9 flagellomeres in males are significantly higher than that of females (Fig 2B). All flagellomeres had similar morphology, but their widths gradually increase from F1 to F9. The amount of sensilla is similar from F1 to F7, while increasing from F8 to F9. Sch3 is found only distribute on F1 of males (Fig 5B and 5F), while all other sensilla types can be found both in males and females, including Sch1 (Fig 4D), Sch2 (Fig 5E), Sb1 (Figs 5H and 6D), Sb2 (Fig 6E), Sb3 (Fig 6F), Str (Fig 6C) and Sco (Figs 5H and 6F). It's worth noting that, in both sexes, Sb1 and Sb3 can only be found on F8 and F9, and Sb2 as well as Str can only be detected on F9 (Table 1).

### Type and morphology of sensilla

#### Böhm bristle (Bb)

Böhm bristle are always straight and located in a slightly concave socket. The surface is smooth with a sharp tip (Fig 3C). No pore has been found on them. The length of Bb ranges from 4.15 to 15.97 μm and the basal diameter is about 1.69 μm (Table 1).

#### Sensilla chaetica (Sch)

Sensilla chaetica are distinguished by longitudinally arranged furrows. They always located in a slightly concave flexible socket and have thick walls. According to their length, location and outer morphology, the sensilla are further divided into three subtypes:

Sch1 are peg like and stand an angle of 50° to the antennal surface (Figs 3E and 4D). Their lengths vary from 9.98 to 39.85 μm, and their basal diameters are about 2.29 μm.

Sch2 are peg or hair like and showed to be longer and stronger than Sch1 (Figs 3F and 5E). Their lengths vary from 52.97 to 103.60 μm, and their basal diameters are about 4.18 μm.

Sch3 are particularly stronger than other sensilla. The sensilla are thorn-shaped with intensely sharp tip and situated in concave socket (Fig 5F) with an average basal diameter of 5.79 μm, and their lengths vary from 31.58 to 45.05 μm.

#### Sensilla basiconica (Sb)

Sensilla basiconica are considerably shorter than Sch, and they are cone-shaped with blunt tips, smooth cuticle, and insert in convex socket. These sensilla are mainly located on the last two segments of *H*. *variegata* antenna. The sensilla are further divided into three subtypes based on their length, location and morphology:

Sb1 are 4.38–7.53 μm long with sharp-tipped pegs, distal longitudinal grooves, and the basal diameters are about 1.96 μm. Their basal sockets are not obvious and slightly higher than the surface (Figs 5H and 6D).

Sb2 are straight with a length ranging from 7.55 to 8.35 μm. They locate on obvious round convex sockets with a basal diameter of 2.18 μm. They are smooth-walled conical pegs with apical nipples (Fig 6E).

Sb3 are longer than the former two subtypes. They have smooth cuticle with blunt tips. In most cases, these sensilla are slightly curved (Fig 6F). They are 11.32–15.31 μm long and about 2.11 μm wide at the base.

#### Sensilla trichodea (Str)

Sensilla trichodea are slender, with smooth surface, locate in round concave socket. These antennal hairs gradually tapered from base to top (Fig 6C). Their lengths vary from 8.23 to 11.39 μm, and the basal diameter is about 1.31 μm.

#### Sensilla placodea (Sp)

Sensilla placodea are elliptical plates with smooth surface, like a small button. They have no obvious socket (Fig 3D). The diameter of them are about 1.2 μm.

#### Sensilla coeloconica (Sco)

Sensilla coeloconica are small pit-organs resembling pores. They have no visible cones in the center of the opening (Figs 3E and 6F). The diameter of the opening is about 0.81 μm.

### Distribution of sensilla

Böhm bristle can only be found on the intersegmental area, i.e. between the head and the scape (Fig 3C), or between the scape and the pedicel (Fig 4C). Sensilla chaetica is the most widely distributed sensilla type on the antenna of *H*. *variegata*. Sch1 and Sch2 are universally distributed on all antennal segments, while only one pair of Sch3 distribute on the terminal part of F1 of the male antennae (Fig 5B). Sb1 can only be detected on the terminal part of last two segments in both sexes, and the quantities are 3–4 on F8 and 5–6 on F9. Sb2 can only be found in the distal area of the apical flagellomere in both sexes. Sb3 often locate beside Sb1 in the similar region of F8 and F9. Sensilla trichodea are located on the tip of F9. Sensilla placodea are rare and only one pair of Sp situated on the scape of the female antennae. Sensilla coeloconica is universal and can be found on all antennal segments in both sexes.

## Discussion

In this work, we have comprehensively revealed the fine morphology of the antennae of *H*. *variegata* and made a comparison of females and males. The morphology and segmentation of *H*. *variegata* antennae are quite similar to other lady beetles reported in former studies (Table 2), except for that the flagellum contain 8 flagellomeres in *Cryptolaemus montrouzieri* [39] and 7 flagellomeres in *Pseudoscymnus tsugae* [40]. From the outer morphology of antennae, there is no obvious difference between the two sexes, while they are relatively stronger in males than in females.

**Table 2:** Segmentation and sensilla type on antennae of lady beetles.

Lady beetles	Segmentation of antennae	Str	Sb	Sch	Sco	Bb	Other sensilla types	Citation
*Semiadalia undecimnotata*	11	1	3		1	1	Chetiform sensilla	Herve et al. 1995
*Hippodamia convergens*	11	1	1			1	Chetiform sensilla	Hamilton et al. 1999
*Pseudoscymnus tsugae*	9		3	2				Broeckling and Salom 2003
*Coccinella septempunctata*	11	1	1	1			Sensilla campaniformia, Sensilla ampucellaceous, Sensilla scolopalia, Sensilla placodea, Hook shaped sensilla	Srivastava and Omkar 2003
*Aiolocaria mirabilis*	11	3	3	3	1	1		Liu et al. 2006
*Harmonia axyridis* (melanic forms)	11	4	4	4	1	1	Sensilla placodea	Chi et al. 2009
*Harmonia axyridis* (succinea forms)	11	4	4	4	1	1	Sensilla sporangium	Chi et al. 2009
*Cryptolaemus montrouzieri*	10	4	4	4	1	1	Sensilla auriciliica, Cavity-like sensilla	Liu et al. 2013
*Propylaea japonica*	11	1	1	1		1	Cavity-like sensilla	Gao et al. 2017

Bb = Böhm bristle; Sch = sensilla chaetica; Sb = sensilla basiconica; Str = sensilla trichodea; Sp = sensilla placodea; Sco = sensilla coeloconica.

The value below each type of sensilla represent the number of subtypes.

### The difference of antennae sensilla between females and males

We have identified and analyzed six types of sensilla, and three subtypes were further identified for Sch and Sb. It is worth noting that Sch3 distributed only on the fixed position of male antennae of *H*. *variegata*. Such sex-dependent sensilla have been also found in other lady beetles. For example, two subtypes of chetiform sensilla are uniquely present on the male antennae of *Semiadalia undecimnotata*, while another one subtype of chetiform sensilla is only present on female [13], and in *Coccinella septempunctata*, a single hook-shaped sensilla is also present on male antenna only [36]. However, in many other species, such as *Hippodamia convergens*, *Pseudoscymnus tsugae* and *Harmonia axyridis*, such sexual dimorphism was not found [4,35,40]. For most insects, the sexual dimorphism in antennae is a ubiquitous phenomenon. In general, the antennae of males are more complex than females because they need more sensilla to detect the sex pheromones in female searching process [1]. Whereas, such differences are usually not present in gregarious insects [35] for the reason that they may use auditory or visual cues to locate the females [8,41]. Based on these, we deduced that the sexual dimorphism of antennal sensilla might be species specific, and the mechanism of specific sensilla in *H*. *variegata* still need more researches to elucidate.

### Sensilla in antennae of different lady beetles

Along with specific searching behaviors, the morphology, ultrastructure, distribution and abundance of sensilla have been adapted in different insects to improve their efficiency and sensitivity of odor perception [18]. Various studies have been conducted on antennae sensilla of lady beetles, and the types are quite different among species (Table 2). In general, sensilla trichodea and basiconica are the basic types that distributed on all reported species. Besides, sensilla chaetica, scoeloconica and Böhm bristle are also universal in majority of species. More than one subtypes can be divided in sensilla trichodea, basiconica and chaetica according to their length, distribution and outer morphology. In addition, some unusual types have also been identified on a few individuals (Table 2). From a purely name point of view, the antennal sensilla exhibit a lot of variation among different species. However, we found that in different species, sensilla with the similar external morphology may be defined as different sensilla type, which suggests that determining a name of sensilla need to be more careful and circumspect. Criterion for universal naming of sensilla should be built as soon as possible.

### Function of antennal sensilla

The Böhm bristles (Bb) on *H*. *variegata* appeared to be nearly identical to those described on *Semiadalia undecimnotata* [13], *Hippodamia convergens* [4], *Harmonia axyridis* [35]; *Cryptolaemus montrouzieri* [39] and *Propylaea japonica* [34]. In most insects, Bb have been demonstrated in the same location and considered as a separate sensilla type [42]. Bb in *H*. *variegata* located in the articulation between the head and scape and scape and pedicel, which is in accord with other studies. Depend on their specific location, Bb are deduced to be mechanoreceptors that perceive antennal movement and position [3,35,43–45].

Sensilla chaetica (Sch) is the most abundant sensilla in not only the antennae but also the mouthpart of *H*. *variegata* [46]. They are also universal on the antennae of many other insects [3,18,35]. Sometimes, this type of sensilla were recognized as sensilla trichodea due to their similar morphology [16]. For many insects, Sch is the longest sensilla on antennae and at a high density, so that they are presumed to sense mechanical stimulus prior to other sensilla types [8]. In *Psylliodes chrysocephala*, Sch is suggested to be contact chemosensilla, which responds to chemicals presented in plant surface waxes when contacts a leaf with antennae [47]. Another study found that Sch were shown to have mechanical reception functions in electrophysiology recordings [18]. From the ultrastructure, Sch is similar to gustatory receptors that have a single pore on the terminal part [48]. In this study, Sch3 are distributed on the distal region of the first flagellomere with their tips toward next flagellomere. This specific location suggested a function for sensing movement and vibration of adjacent segments [49]. Besides, they may contribute to the detection of sex pheromones in female searching process due to the fact that they are only be found on male antennae.

Sensilla basiconica (Sb) mainly distribute on the terminal part of flagellum with a small number. In view of the special location, they may be the first to contact external substance immediately, so they were assumed to serve a contact chemoreceptive function [9,40,50]. On the other hand, Sb are interpreted to have an olfactory function [9,48,51,52]. For example, they were considered to play critical roles in odor sensing for searching suitable habitat and food resources in *Callosobruchus chinensis* [3] and *Tetrigus lewisi* [49]. Besides, Sb are presumed to be sensible of sex pheromone because greater number of Sb are found in male versus female antennae of *Leptura aethiops* [53] and *C*. *chinensis* [54]. Sb are also hypothesized to be the receptors of possible plant volatile in *Harmonia axyridis* [35] and *Phoracantha semipunctata* [51].

Sensilla trichodea (Str) are common types in antennae of many lady beetles and densely distributed on the tip of flagellum [4,35–36]. In this study, only one subtype of Str can be found on the antennae of *H*. *variegata*, which are relatively less than other lady beetles [35–36,39]. Studies showed that *Hippodamia convergens* no longer responded to odors when their Str were removed. Based on this phenomenon, the Str are deduced to be responsible for long-range olfactory reception [4]; Chi assumed that Str may be the pheromone receptors account for the aggregative behavior of *H*. *axyridis* [35]. In insects of Orthoptera, Str are quite common and have several subtypes. They were described as olfactory receptor neurons to host plant odors and aggregation pheromone in *Rhynchophorus palmarum* [55], and as sex or aggregation pheromone receptors in *Bembidion properans* [44].

Sensilla placodea (Sp) are rare and only located on the scape of *H*. *variegata*. Up to now, in Coccinellidae, they have only been found on the same position in the melanic forms of *H*. *axyridis* [35]. The exact function of these sensilla are not clear, but may be related to the reception of pheromone and plant volatile [35].

Sensilla coeloconica (Sco) in *H*. *variegata* are small pit organs, which are similar to that of other beetles, such as *H*. *axyridis* [35]. However, this type of sensilla may be given other name, such as sensilla cavity in *Aiolocaria mirabilis* [37], *Cryptolaemus montrouzieri* [39] and *Propylaea japonica* [34]. Similarly, the sensilla cavity in *Callosobruchus chinensis* [3] and sensilla ampucellaceous in *C*. *septempunctata* [36] have the same morphology with Sco in this study. These sensilla have been reported to be sensitive to water vapors, carbon dioxide, and thermal changes [56].

In conclusion, the differences in shape are not always in accord with differences in functionally relevant internal structures. Thus, it is not enough to determine a name of sensilla relying on morphology alone [46]. Confirmation of sensilla function requires not only external morphology, but also more investigation on structural properties of functional relevance [38]. Moreover, the behavior analysis should also be taken into account.

### Antennal sensilla versus host preference

The relationships of antennal sensilla and host preference have been studied in several insect orders. In the study of six Triatominae species (Heteroptera), the number and distribution of four antennal sensilla types was found to vary according more to habitat type than taxonomic status [57]. Another study on wasps (Hymenoptera: Philanthinae), including species that hunt exclusively either on beetles or on bees to feed their larvae, found that grooved peg sensilla can only be found in three bee-hunting species [58]. In addition, the cluster analysis confirmed that the presence, density, size and distribution of certain sensilla should be determined by the prey type [58]. This idea was coincidence with another study. In biting midges (Diptera: Ceratopogonidae), researchers found the numbers of specific sensilla types (sensilla trichodea, sensilla coeloconica and sensilla basiconica) are significantly higher in ornithophilic species than mammalophilic ones, and the opportunistic species have intermediate numbers of these sensillum types [59]. They deduced that these differences in the sensilla number are strongly correlated with host preference and not with phylogeny [59]. However, in another study, the sensilla densities are very similar in two anopheline sibling species (Diptera) with different host preferences [60]. In Coleoptera, relevant studies were much rare, while the role of mouthpart sensilla in feeding process have been studied in several species [46,61,62]. We found that many types of sensilla are distributed both on antennae and mouthpart, thus more comparative studies should be done to conform if there is correlation between the number of sensilla and feeding preference.
